# Recurrent Gallbladder Carcinoma With pMMR/MSS Achieved a Complete Response Following Camrelizumab Combined With Apatinib: A Case Report

**DOI:** 10.3389/fonc.2021.783158

**Published:** 2022-01-13

**Authors:** Liting Zhong, Xiaoyu Liu, Zelei Li, Xuebing Zhang, Yuli Wang, Weiwei Peng

**Affiliations:** ^1^ Department of Oncology, Ganzhou People’s Hospital (The Affiliated Ganzhou Hospital of Nanchang University), Ganzhou, China; ^2^ Department of Imaging, Ganzhou People’s Hospital (The Affiliated Ganzhou Hospital of Nanchang University), Ganzhou, China

**Keywords:** gallbladder carcinoma, PD-1 blockade, anti-angiogenic therapy, pMMR/MSS, case report

## Abstract

Gallbladder carcinoma (GBC) with proficient mismatch repair (pMMR)/microsatellite stable (MSS) is associated with limited response to programmed death-1 (PD-1) inhibitor monotherapy. Limited data of PD-1 blockade combined with anti-angiogenic therapy in GBC are reported. One recurrent GBC patient with pMMR/MSS was treated with camrelizumab plus apatinib. After 4 cycles of combination therapy, the patient achieved a durable complete response with manageable toxicity. The next-generation sequencing and immunohistochemistry analysis showed that tumor mutation burden (TMB) was 7.26 mutants/Mb and PD-L1 expression was 10% (tumor proportion score) and 20% (immune proportion score). This case suggests that camrelizumab in combination with apatinib may be an effective treatment option for GBC patients with pMMR/MSS status, who have moderate expression of TMB and PD-L1. Additionally, TMB and PD-L1 expression may serve as potential biomarkers for predicting PD-1 inhibitor response of GBC. Furthermore, this needs to be verified in future studies.

## Introduction

Gallbladder carcinoma (GBC) is one of the most common primary malignancies of the biliary tract. Treatment protocols for advanced GBC are limited, and the prognosis is extremely poor, with a median overall survival of around 4–7 months (1). At present, combined chemotherapy is the main treatment for patients with advanced GBC; however, this therapy is highly toxic and provides patients limited survival benefit. Moreover, pembrolizumab was approved for advanced GBC patients with deficient mismatch repair (dMMR) or microsatellite instability-high (MSI-H). However, dMMR/MSI-high cancer is extremely rare. More effective and safe treatments are urgently needed for recurrent GBC, especially those with proficient mismatch repair (pMMR)/microsatellite stable (MSS) status.

Immunotherapy such as immune checkpoint inhibitors (ICIs) had become prospective therapeutic schemes in a variety of cancers. Several clinical trials were also conveyed in biliary tract cancers (BTC), including the KEYNOTE-028 trial and KEYNOTE-158 trial. However, the objective response rate (ORR) of ICI monotherapy are only about 6%–13% in BTC (2). Therefore, a new scheme of treatment combination is needed to overcome ICI resistance. Camrelizumab, a high-affinity, humanized, IgG4-κ PD-1 monoclonal antibody, was widely applied for cancer therapy. Apatinib, a selective vascular endothelial growth factor receptor (VEGFR)-2 inhibitor, could modulate tumor microenvironment (TME) *via* relieved hypoxia, accelerated tumoral infiltration of CD8+ T cells, and decreased recruitment of tumor-associated macrophages (3). For advanced HCC, camrelizumab and apatinib combination therapy acquired a 34.3% ORR as the first-line and 22.5% as the second-line therapy (4). Moreover, several trials for osteosarcoma, gastric cancer, and advanced triple-negative breast cancer have shown that camrelizumab combined with apatinib was effective and tolerable (5–7). However, data of clinical trials of camrelizumab plus apatinib in GBC patients are insufficient.

Here, we report a recurrent metastatic GBC patient with pMMR/MSS who has obtained complete response to camrelizumab combined with apatinib treatment.

### Case Presentation

The patient was a 60-year-old male with a medical history of hypertension and hepatitis B cirrhosis. He received regular anti-hypertension and antiviral treatment. In May 17, 2020, abdominal computed tomography (CT) revealed a gallbladder mass at the fundus (2.7 × 2.2 cm in size) at an annual medical examination. Enhanced scan showed that the mass was markedly enhanced. Enlarged lymph nodes were not found in the abdomen. He showed no obvious symptoms of discomfort, and physical examination was normal. Then, he received open cholecystectomy and regional lymphadenectomy in May 27, 2020. Surgeons found that the tumor directly invaded the liver. Therefore, hepatectomy with partial resection of segments 4 and 8 was performed. Postoperative pathology indicated a pT3N0M0 adenocarcinoma with poor differentiation, without lymph node metastasis and with a negative surgical margin. The abundance of ERBB2 mutation was 26.94%, which suggested the possible therapeutic potential of trastuzumab. Then, he was treated with 6 adjuvant treatments from July 18, 2020, to October 18, 2020. Gemcitabine (1,000 mg/m^2^, d1, d8), albumin-bound paclitaxel (125 mg/m^2^, d1, d8), and trastuzumab (6 mg/kg, d1) were administered every 3 weeks.

**Figure 1:**
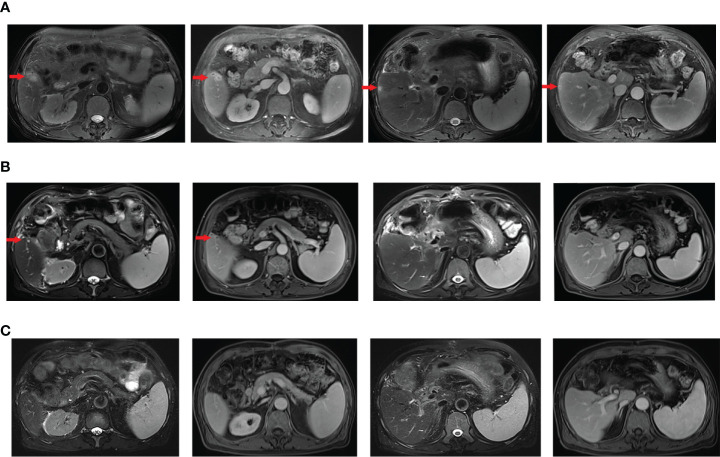
Enhanced abdominal magnetic renounce imaging (MRI) of the reported case. **(A)** T2-weighted and arterial phase images of liver metastasis before camrelizumab combined with apatinib (November 6, 2020). The red arrows direct to the recurrent lesions in the liver. **(B)** MRI showed a partial response in liver metastasis from T2-weighted and arterial phase images after 2 cycles of combination therapy (December 22, 2020). The mass (arrow) adjacent to the surgical zone decreased to 0.6 cm in the maximum diameter (1.1 cm before treatment). The lesion under the liver capsule of S5 disappeared. **(C)** MRI showed that lesions in the liver completely disappeared after 4 cycles of combination therapy (February 1, 2021).

**Figure 2:**
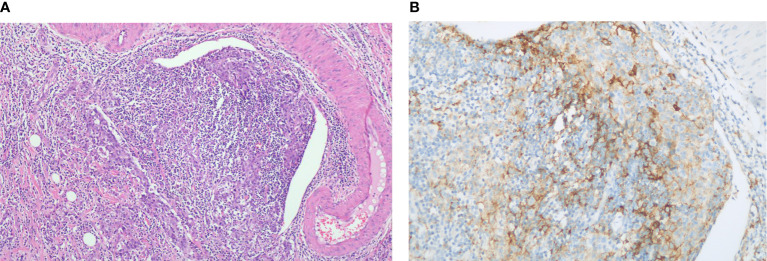
Histopathology of gallbladder carcinoma. **(A)** H&E stain, original magnification ×100. **(B)** PD-L1 immunohistochemistry (antibody Ventana SP263), original magnification ×200.

In November 6, 2020, abdominal magnetic resonance imaging (MRI) indicated multiple lesions in the right lobe of the liver, the largest measuring 1.1 cm × 1.1 cm adjacent to the surgical zone (**Figure 1A**). Moreover, the carbohydrate antigen 19-9 (CA-199) level was elevated at 29.03 U/ml (normal levels, <25 U/ml). This patient refused chemotherapy. Additionally, he received next-generation sequencing (NGS) analysis of tissue sample. NGS was performed on an Illumina HiSeq sequencer (Illumina, San Diego, CA) with a median unique exon coverage depth of 800× in a College of American Pathologists (CAP) and Clinical Laboratory Improvement Amendments (CLIA) certified laboratory (3D Medicine Inc., Shanghai, China). NGS results are presented in **Table 1**. The patient had tumor mutation burden (TMB) of 7.26 mutants/Mb, with pMMR/MSS status. Immunohistochemistry (IHC) results showed that programmed death ligand-1 (PD-L1) expression was 10% (tumor proportion score, TPS) and 20% (immune proportion score, IPS; **Figure 2**). The profile of genetic alterations included ERBB2, AR, TERT, ERBB3, KMT2C, BTK, and TP53. Consequently, and after obtaining the consent from the patient, camrelizumab combined with apatinib was initiated from November 16, 2020. He was administered 200 mg camrelizumab intravenously over 30 min every 3 weeks. The patient received apatinib orally at 500 mg per day. One month later, he developed grade 3 hypertension, which was attributed to apatinib. Then, the dose of apatinib was decreased to 250 mg per day. In the following treatment, no serious adverse event was observed except for grade 2 leukopenia and grade 2 thrombocytopenia, which may be attributed to both drugs and cirrhosis with splenomegaly.

**Table 1:** The results of next-generation sequencing (NGS) analysis.

Cohort	Value
Microsatellite	Microsatellite stable
MLH1, MSH2, MSH6, PMS2	No variants detected
Tumor mutation burden	7.26 Mutants/Mb
PD-L1 expression	Positive, 10% (tumor proportion score *^a^*), 20% (immune proportion score *^b^*)
EBV	Negative
HLA Class I	Partial homozygous
Gene	Mutation (abundance/copy number)
ERBB2	p.V777L Exon 20 (26.94%)
AR	Increase in copy number (6)
TERT	c.-124C>T (7.42%)
ERBB3	p.G284R Exon 7 (11.72%)
KMT2C	p.I3493Pfs*13 Exon 43 (6.31%)
BTK	Increase in copy number (6)
TP53	p.R306*Exon 8 (11.21%), p.C135Ffs*15 Exon 5 (4.62%)

a Tumor proportion score (TPS) was defined as the percentage of tumor cells showing a membranous PD-L1 expression with any intensity over all tumor cells.

b Immune proportion score (IPS) was defined as the percentage of PD-L1-positive immune cells of any intensity over all tumor-associated immune cells.

**Figure 3:**
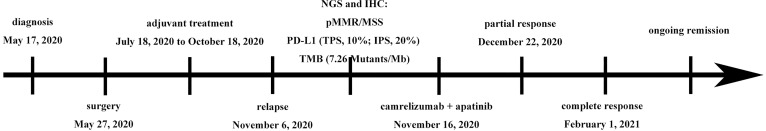
Timeline of the clinical course in this patient.

On December 22, 2020, MRI showed exceptional shrinkage of the tumor and the lesion under the liver capsule of segment 5 (S5) disappeared, with a partial response after two cycles of treatment (**Figure 1B**). Furthermore, lesions in the liver completely disappeared (**Figure 1C**) and CA-199 was reduced to normal levels (22.93 U/ml) after four cycles of treatment. The therapeutic effect was determined to be a complete response, as assessed by the Response Evaluation Criteria in Solid Tumors RECIST Version 1.1. At the last follow-up (September 24, 2021), the patient was in good health and MRIs confirmed a sustained response. Since initiating camrelizumab and apatinib, he has obtained a progression-free survival (PFS) of 10 months. The patient is still on treatment with camrelizumab (200 mg, Q3 weeks) plus apatinib (250 mg QD) as of the submission date of this article. **Figure 3** outlines the patient’s history.

## Discussion

There are limited treatment options for patients with recurrent GBC. Herein, we report a case in which camrelizumab combined with apatinib is effective and safe in a recurrent GBC patient with pMMR/MSS status. This is the first report demonstrating that recurrent GBC with pMMR/MSS has achieved a complete response following PD-1 inhibitor plus anti-angiogenic therapy.

Several studies have demonstrated that ICI and anti-angiogenic agent combination therapy has synergistic antitumor activity in solid tumors (8–10). The vascular endothelial growth factor (VEGF) has pleiotropic immunosuppressive effects including impairment of dendritic cell function, as well as mobilization of immunosuppressive cells such as myeloid-derived suppressor cells, regulatory T cells, and tumor associated macrophages (11). Currently, most trials combining anti-VEGF therapy with immunotherapy for GBC are in the recruitment period. In addition, few trials have reported detailed outcome. The initial result of combining camrelizumab with apatinib in 21 advanced BTC patients has shown that no patient achieved complete response, 4 patients obtained partial response, and 11 patients had stable disease with manageable toxicity (12). The synergistic effect of anti-angiogenic agents with immunotherapy may be largely attributed to the immunomodulatory function of anti-angiogenic agents on TME rather than their direct antitumor activity (13, 14), as it was found that a low-dose anti-angiogenic agent was superior to higher doses in inhibiting tumor growth in animal models when combined with immunotherapy (3). In our case, the patient with reduced dose of apatinib (250 mg, QD) was effective. Hence, future clinical trials were needed to validate the optimal dosage of apatinib considering side effects and clinical response.

Recently, the U.S. Food and Drug Administration granted approval to pembrolizumab for BTC with dMMR/MSI-high. However, dMMR/MSI-high cancer is extremely rare, seen in only 1% to 3% of patients with BTC (15, 16), suggesting the necessity of identifying better predictive biomarkers for immunotherapy. Several studies have shown that PD-L1 expression is associated with the efficacy of PD-1 blockade (17, 18). Currently, a positive rate of PD-L1 expression of between 9% and 11.6% was reported in BTC patients receiving ICIs (19, 20). Kim et al. indicated that the PD-L1 expression rate of tumor cells (≥1%) is correlated with better ORR and prolonged PFS in a study focusing on nivolumab treatment for refractory BTC (21). In the KEYNOTE-158 trial, the patients with PD-L1-expressing BTC had an ORR 6.6%, and the ORR was 2.9% in patients with PD-L1 non-expressing tumors (2). PD-L1-expressing tumors were defined as a combined positive score of ≥1, which was calculated as the number of PD-L1–positive cells (tumor cells, lymphocytes, macrophages) divided by the total number of tumor cells and multiplied by 100. TMB may be the next predictor for PD-1 inhibitor therapy (22). It has been demonstrated that cancers with a high TMB express higher numbers of neoantigens, which can be identified by the immune system in response to ICI treatment (23–25). In our case, the patient with pMMR/MSS status had moderate expression of PD-L1 (TPS, 10%; IPS, 20%) and TMB (7.26 mutants/Mb). Camrelizumab combined with apatinib induced successful results in the recurrent GBC patient with pMMR/MSS. Therefore, it may be valuable to assess PD-L1 expression and TMB for the GBC patients, especially those with pMMR/MSS status, in order to direct the adjuvant treatment.

We reported that a recurrent GBC patient with pMMR/MSS achieved durable complete response from the combined treatment of camrelizumab and apatinib. PD-L1 expression and TMB may be the predictive markers for GBC patients who would profit from anti-PD-1 therapy. Future studies on combination therapy will reveal the true potential of combination ICIs and anti-angiogenic agents in GBC.

## Data Availability Statement

The datasets presented in this study can be found in online repositories. The names of the repository/repositories and accession number(s) can be found in the following: NCBI SRA BioProject, accession no: PRJNA786280.

## Ethics Statement

Written informed consent was obtained from the individual(s) for the publication of any potentially identifiable images or data included in this article.

## Author Contributions

LZ: manuscript writing. XL and ZL: imaging analysis. XZ and YW: data collection. WP: manuscript revision. All authors contributed to the article and approved the submitted version.

## Funding

This work was supported by the Health Science and Technology Project of Jiangxi Province (202140765).

## Conflict of Interest

The authors declare that the research was conducted in the absence of any commercial or financial relationships that could be construed as a potential conflict of interest.

## Publisher’s Note

All claims expressed in this article are solely those of the authors and do not necessarily represent those of their affiliated organizations, or those of the publisher, the editors and the reviewers. Any product that may be evaluated in this article, or claim that may be made by its manufacturer, is not guaranteed or endorsed by the publisher.
